# Liver test abnormalities predict complicated disease behaviour in patients with newly diagnosed Crohn’s disease

**DOI:** 10.1007/s00384-016-2706-3

**Published:** 2016-11-29

**Authors:** Jessika Barendregt, Myrthe de Jong, Jeoffrey J. Haans, Bart van Hoek, James Hardwick, Roeland Veenendaal, Andrea van der Meulen, Nidhi Srivastava, Rogier Stuyt, Jeroen Maljaars

**Affiliations:** 1 Department of Gastroenterology-Hepatology, Leiden University Medical Centre, Leiden, The Netherlands; 2 Department of Gastroenterology-Hepatology, Maastricht University Medical Centre, Maastricht, The Netherlands; 3 Department of Gastroenterology-Hepatology, Haaglanden Medical Centre, The Hague, The Netherlands; 4 Department of Gastroenterology-Hepatology, Haga Hospital, The Hague, The Netherlands

**Keywords:** Inflammatory bowel disease, Prognosis, Complications

## Abstract

**Backgrounds:**

In coeliac disease, the prevalence of liver test abnormalities (LTAs) is higher in patients with more severe mucosal inflammation. In Crohn’s disease, prognosis is related to the severity of mucosal inflammation.

**Aim:**

The aim of this study was to investigate whether the presence of LTA predicts the occurrence of complicated disease behaviour in newly diagnosed Crohn’s disease.

**Methods:**

A retrospective cohort study was performed in patients newly diagnosed with Crohn’s disease between 2002 and 2011. The complicated disease was defined as the occurrence of stricturing and/or perforating disease. LTAs were defined as a value of any of alkaline phosphatase (AP), gamma-glutamyl transpeptidase (GGT), aspartate aminotransferase (AST), or alanine aminotransferase (ALT) over the upper limit of normal.

**Results:**

Three hundred eighty-three patients were included, of whom 34.1% had LTA. LTAs were mostly mild (less than two times the upper limit of normal). During the 5-year follow-up, 33.1% of patients in the group with LTA developed complicated disease behaviour compared to 14.6% in patients without LTA (*p* < 0.001). The presence of LTA was identified as a risk factor for complicated disease behaviour (HR 2.6, 95% confidence interval (CI) 1.5–4.2, *p* < 0.0001).

**Conclusions:**

In newly diagnosed Crohn’s disease, the presence of LTA was an independent risk factor for the development of complicated disease behaviour.

**Electronic supplementary material:**

The online version of this article (doi:10.1007/s00384-016-2706-3) contains supplementary material, which is available to authorized users.

## Introduction

Liver test abnormalities (LTAs) are frequently observed in patients with inflammatory bowel disease (IBD), affecting between 3 and 50% of patients [1, 2]. In IBD patients, LTAs are most frequently ascribed to the use of medication or the presence of hepatic steatosis [3]. For instance, Gisbert et al. followed 786 IBD patients for 5 years and found LTA in 15% of patients, of which 43% were ascribed to thiopurines and hepatic steatosis was considered the cause in 41% of patients [3].

Whether liver test abnormalities have any clinical significance has recently been studied. Morsy and Hasanain found a relation between clinical and endoscopic indices of severity and levels of alkaline phosphatase (AP), alanine aminotransferase (ALT), and aspartate aminotransferase (AST) [15]. Mendes et al. demonstrated that inflammatory bowel disease patients with any LTA had a worse survival compared to patients without LTA [2]. A similar relation has been observed in the general population [4]. However, whether the presence of LTA has any prognostic value with regard to disease behaviour in patients with Crohn’s disease (CD) has not been studied. Recently, a study in patients with newly diagnosed coeliac disease demonstrated a relationship between the severity of mucosal inflammation and the presence of liver test abnormalities (AST, ALT, and gamma-glutamyl transpeptidase (GGT)) [5]. Although the pathogenesis of inflammation in coeliac disease has a different pathogenesis form of the inflammation observed in CD, there are also similarities, such as an increased bowel permeability. In CD, prognosis is related to the severity of mucosa inflammation [6]. Therefore, we hypothesized that in newly diagnosed CD, the presence of LTA has a prognostic value with regard to the development of complicated disease behaviour. We therefore retrospectively studied the relationship of LTA with complicated disease behaviour in a cohort of all CD patients newly diagnosed between 2004 and 2011 in one tertiary referral hospital and two large teaching hospitals.

## Materials and methods

### Study population

A retrospective study was performed in CD patients newly diagnosed between 1 January 2002 and 31 December 2011 at the Leiden University Medical Centre (Leiden), a tertiary IBD referral centre, and the Haga Hospital (The Hague) and the Haaglanden Medical Centre (The Hague), two large teaching hospitals. Patient charts were obtained from an electronic patient database, using (CD) diagnosis code “601”.

In order to be included, patients had to be newly diagnosed with CD between 1 January 2002 and 31 December 2011, the diagnoses must have been made in one of the three hospitals, and laboratory evaluation including ALT, AST, AP, GGT, and C-reactive protein (CRP) had to be available for analysis.

Patient characteristics such as demographics (age, gender, weight, height), medical history, medication, intoxications, and medical examinations (laboratory evaluation, ultrasound, viral evaluation, auto-immune evaluation) were retrieved from the medical charts. Data on the disease course were collected from medical charts. These included the use of steroids, 5-ASA, immunosuppressive treatment, anti-TNF, hospitalization, complications, surgery, liver and biliary diseases, and mortality during the disease course of CD. CD was classified using the Montreal classification [7].

### Timing of lab tests

Although in most cases multiple laboratory test results were available, only the results of the first visit to the outpatient clinic or emergency department were used. As these lab tests were taken prior to the IBD diagnosis, or coincided with the moment of diagnosis, these results were all obtained prior to the initiation of any IBD therapy.

### Definitions

The diagnosis of Crohn’s disease was made on the basis of standard clinical, endoscopic, radiological, and pathological criteria [8]. The relevant liver tests were AP, GGT, AST, and ALT. Liver test abnormalities were defined as an elevation of any of these liver tests above the upper limit of normal. During the study period, different upper limits of normal were used for the different liver tests at different moments in the participating hospitals. We therefore expressed all liver test values in this paper as the quotient of the test result divided by the upper limit of normal at that moment. An *R* value was calculated to assess whether liver test abnormalities were predominantly cholestatic, hepatocellular, or mixed [9]. The *R* value was defined as the serum alanine aminotransferase/upper limit of normal (ULN) divided by the serum alkaline phosphatase/ULN. The value *R* ≥ 5 is labelled as hepatocellular, *R* < 2 is labelled as cholestatic, and 2 < *R* < 5 is labelled as “mixed”.

### Study outcome

The primary endpoint of this study was complicated disease behaviour, defined as the occurrence of a stenosis, abscess, fistula, or perforation, during the first 5 years of follow-up after diagnosis. Other endpoints were hospitalizations and surgery. In patients with complicated disease behaviour at diagnosis (B2 and/or B3, according to the Montreal classification), follow-up ended at the next occurrence of a complication. Hospitalization was defined as admission to the hospital specifically due to an exacerbation and/or complaints of CD. Surgery was defined as the need for surgical intervention due to complications or severe complaints of CD.

### Statistical methods

Data were analysed using SPSS 20.0. To compare baseline patient characteristics of the groups with and without LTA at diagnosis, a chi-square test was used for categorical variables, whereas an independent samples *t* test was used for continuous variables. If the *p* value was ≤0.05, the results were considered statistically significant.

Survival analysis was performed using the Kaplan-Meier analysis with log-rank test comparing patients with and without liver test abnormalities. Other variables associated with a complicated disease course were found using univariate Cox regression analysis. Variables with *p* value ≤0.1 in univariate analysis were used for multivariate Cox regression analysis, in which hazard ratios (HRs) were calculated.

## Results

### Study population

A total of 383 patients were included in the study (Table 1). Of these patients, 62% were female. One patient with chronic liver disease (small duct Primary Sclerosing Cholangitis (PSC)) was excluded. Of the 383 patients included in this study, 131 had LTA (34.1%). Patients with LTA were older than those without LTA and had a higher CRP. Groups were similar with regard to the Montreal classification.

**Table 1:** Patient characteristics

	All patients (*n* = 383)	LTA (*n* = 131)	No LTA (*n* = 252)
Female, *n* (%)	240 (62.7)	85 (62)	155 (65)
BMI in kg/m^2^ (*n* = 202), *n* (%)			
<18.5	44 (11.5)	16 (24.6)	28(20.4)
18.5–26	107 (27.9)	34 (54.7)	73 (49.2)
>26	51 (13.3)	16 (24.8)	35 (26.2)
Mean BMI in kg/cm^2^ (SD; IQR)	23.3 (5.9; 19.5–26.0)	23.3 (6.0; 19.0–26.1)	23.4 (5.8; 19.5–25.9)
Features of metabolic syndrome^a^, *n* (%)	54 (14.1)	22 (16.8)	32 (12.7)
Use of non-IBD co-medication, *n* (%)	227 (59.3)	81 (59.6)	146 (59.1)
No comorbidity, *n* (%)	261 (68.1)	91 (66.9)	170 (68.8)
Other auto-immune disease, *n* (%)	23 (6)	9 (5.6)	14 (6.9)
Mean CRP at diagnosis in mg/L (SD; IQR)	56 (68; 10–78.5)	73.6 (77.6; 18–115.5)*	46.7 (61.1; 8–56)*
CRP in mg/L, *n* (%)		**	**
<16		24 (20.5)	83 (41.0)
16–58		42 (39.6)	64 (31.5)
>58		51(47.7)	56 (27.6)
Age at diagnosis, *n* (%)			
A1	33 (8.6)	10 (7.6)	23 (9.1)
A2	222 (58)	68 (30.6)	154 (61.1)
A3	128 (33.4)	53 (40.5)	75 (29.8)
Mean age at diagnosis in years (SD; IQR)	34.2 (16.8; 21–45)	36.7 (17.4; 23–49)*	32.9 (16.5; 20.3–43)*
Location at diagnosis, *n* (%)			
Colonic	97 (25.3)	32 (24.4)	65 (25.8)
Ileal only	129 (33.7)	41 (31.3)	88 (31.8)
Ileocolonic	153 (39.9)	55 (35.9)	98 (38.9)
Upper GI disease	4 (1)	3 (2.3)	1 (0.4)
Behavior at diagnosis, *n* (%)			
B1	281 (73.4)	93 (71.0)	188 (74.6)
B2	77 (20.1)	26 (19.8)	51 (20.2)
B3	25 (6.5)	12 (9.2)	13 (5.3)
Perianal involvement at diagnosis, *n* (%)	39 (10.2)	13 (9.9)	26 (10.3)
Smoking, *n* (%)	137 (35.8)	47(34.1)	90 (47)
Alcohol, *n* (%)			
No alcohol	162 (61.4)	51 (59.3)	111 (62.4)
Use	95 (36)	30 (34.9)	65 (36.5)
Abuse	7 (2.7)	5 (5.8)	2 (1.1)
Liver test abnormalities at diagnosis, *n* (%)	131 (34.2)		
Complication during follow-up, *n* (%)	81 (21.1)	44 (33.6)**	37 (14.7)**
Type of complication, *n* (%)			
Stenosis	56 (18)	32(24.4)**	24 (9.5)**
Abscess	38 (9.9)	13 (9.9)	14 (5.5)
Fistula	36 (9.4)	11 (8.4)	15 (5.9)
Perforation	6 (1.6)	4 (3.1)	2 (0.8)
Hospitalization, *n* (%)	161 (42)	69 (52.7)*	92 (36.5)*
Surgery, *n* (%)	89 (23.2)	37(28.2)	52 (20.6)
Type of surgery, *n* (%)		*	*
Ileocecal resection	63 (16.4)	23 (17.7)	40 (15.9)
Colectomy	13 (3.4)	5 (3.81)	8 (3.1)
Small bowel resection	7 (1.8)	6 (4.5)	1 (0.4)
Other	6 (1.6%)	3 (2.3%)	3 (1.1%)
Medication used during follow-up, *n* (%)			
Prednisone	244 (63.7)	85 (64.9)	159 (63.1)
Mesalamine	174 (45.5)	60 (45.8)	114 (45.2)
Immunomodulator	338 (88.3)	118 (90.1)	220 (87.3)
Anti-TNF	193 (50.4)	71 (54.2)	122 (48.4)

A chi-square test was used for categorical variables, and an independent samples *t* test was used for continuous variables. A1 = age at diagnosis <16 years; A2 = age at diagnosis between 17 and 40 years; A3 = age at diagnosis >40 years; B1 = non-stricturing, non-penetrating disease; B2 = stricturing disease; B3 = penetrating disease

*LTA* liver test abnormalities, *SD* standard deviation, *IQR* interquartile range, *BMI* body mass index, *CRP* C-reactive protein

*p < 0.05, for LTA vs. no LTA; **p < 0.001, for LTA vs. no LTA

^a^Features of metabolic syndrome: diabetes, hypercholesterolemia, and hypertension

When patients were divided into three groups based on their CRP, the prevalence of LTA was highest (48.6%) in the group with the highest CRP (>58 mg/L) compared to 39.6% in the 16–58 mg/L group and 22.4% in the <16 mg/L group (*p* < 0.0001). Patients with an elevated level of AP or GGT most often had a CRP >16 mg/L (for AP, 54/66 (*p* = 0.012); for GGT, 62/74 (*p* = 0.001)).

In general, LTAs were mild (Suppl. Table 4), and in 54% of patients with LTA, only a single liver test was elevated (Suppl. Table 5). GGT was elevated most often (Suppl. Table 6). The *R* ratio was calculated for all patients with LTA: in 79 patients (60%), the *R* ratio was <2 (cholestatic); in 39 patients (29.8%), the *R* ratio was between 2 and 5 (mixed); and in 3 patients (2.3%), the *R* ratio was >5 (hepatocellular).

Thirty-two percent of patients had any comorbid disease, and 6% had an additional auto-immune disorder; these proportions did not differ between patients with and without LTA. No difference was observed with regard to the presence of overweight, diabetes, or other features of the metabolic syndrome. There was also no relationship between the *R* ratio and the presence of overweight, diabetes, or other features of the metabolic syndrome.

Sixty-four percent of patients underwent abdominal ultrasound (Suppl. Table 4; 64.8% in the LTA group vs. 64.7% in the group without LTA), and the prevalence of steatosis was similar between groups. Similarly, no significant differences were found between the prevalence of biliary stones, sludge, hepatomegaly, or gallbladder polyps or adenomyomatosis. Given the mild LTA, a full analysis was not performed in all cases, but in the patients with evaluation for viral or auto-immune liver disease, this evaluation was negative (Suppl. Table 4).

Interestingly, 28 out of the 39 patients with perianal disease at diagnosis were started on anti-TNF compared to 165 out of the 344 patients without perianal disease (72 vs. 48%, *p* = 0.005).

### Relation between the presence of LTA at diagnosis and the disease course of Crohn’s disease

In total, 27% of patients developed complicated disease behaviour during 5 years of follow-up. Twenty-three percent had surgery for CD, and 42% of patients were hospitalized during the first 5 years of follow-up after diagnosis.

In patients with LTA, 33.6% developed complicated disease behaviour, compared to 14.7% of the patients without LTA (*p* < 0.001; Table 1). Similarly, patients with LTA were significantly more often hospitalized. Although the number of patients undergoing surgery did not differ between groups, the type of surgery did differ between groups: more often, a small bowel resection was performed in LTA patients.

Suppl. Table 4 shows the relationship between each liver test and the risk factor for developing complicated disease behaviour, hospitalization, and surgery. Whereas GGT was elevated most often, an elevated level of AP was associated with an increased risk of complicated disease or hospitalization. In Suppl. Table 5, we show that the risk for patients with two abnormal liver tests of developing complicated disease behaviour was higher than that for patients with one abnormal test. However, the presence of more than two abnormal tests did not further increase the risk.

**Fig. 1:**
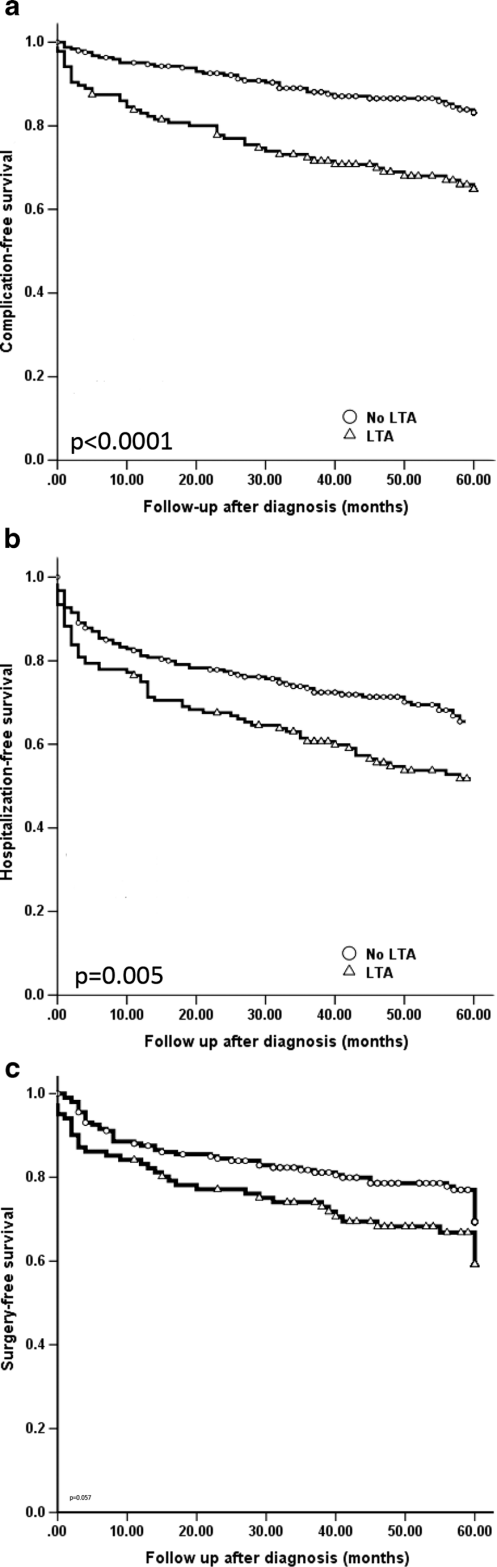
Occurrence of complications, hospitalization, and surgery, when compared between patients with and without liver test abnormalities. **a** Complication-free survival; *p* < 0.0001 between patients with and without LTA (log rank). **b** Hospitalization-free survival; *p* = 0.005 between patients with and without LTA (log rank). **c** Surgery-free survival; *p* = 0.057 between patients with and without LTA (log rank). *LTA* liver test abnormalities

**Fig. 2:**
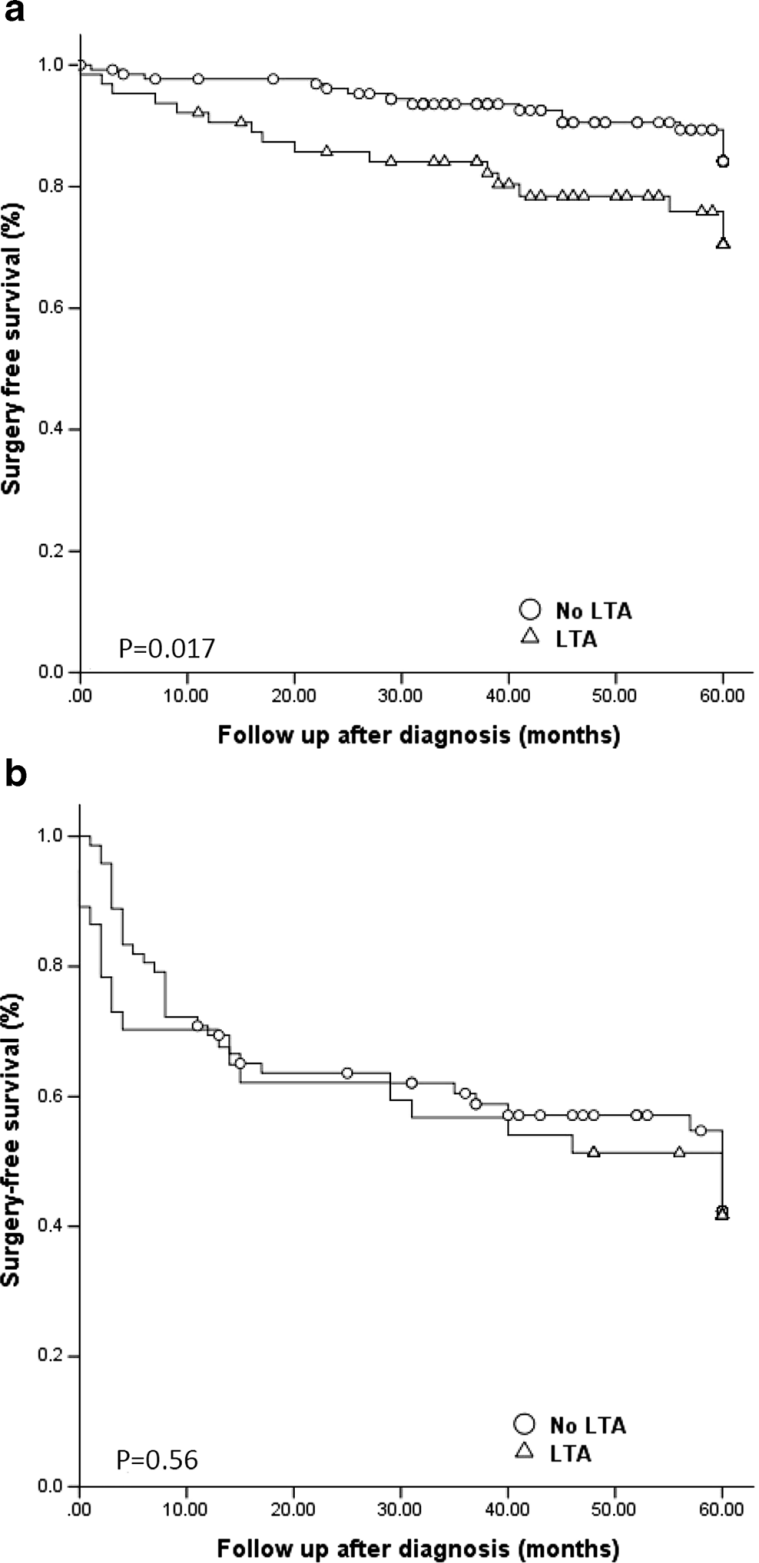
Need for surgery, based on the presence of complicated disease behaviour at diagnosis. **a** Patients without complicated disease at diagnosis; *p* = 0.017 between patients with and without LTA (log rank). **b** Patients with complicated disease at diagnosis; *p* = 0.56 between patients with and without LTA (log rank)

As shown in Fig. 1a, both complication-free survival and hospitalization-free survival were significantly longer in patients without LTA compared to patients with LTA (log rank: *p* < 0.0001; log rank: *p* = 0.005, respectively; Fig. 1b). When all patients were included, the difference for surgery-free survival was not significant (log rank: *p* = 0.057, Fig. 1c), but when only patients with B1 behaviour at diagnosis were analysed, patients with LTA had a shorter surgery-free survival (log rank: *p* = 0.017; Fig. 2).

**Fig. 3:**
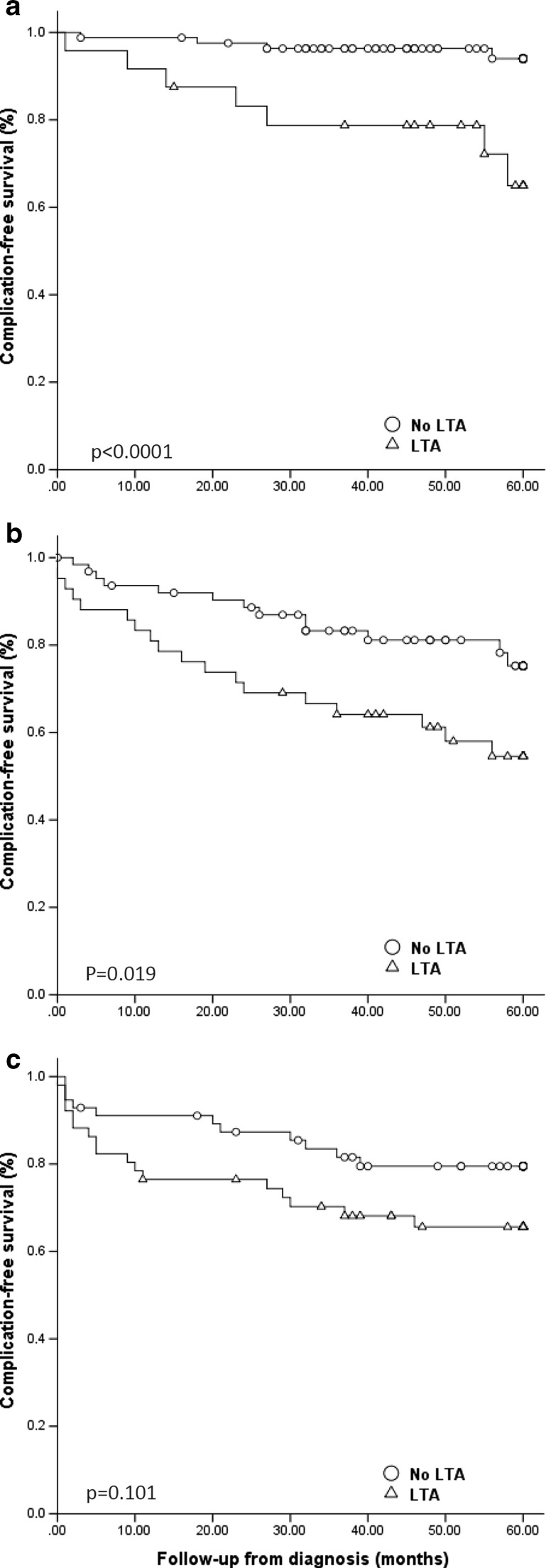
Development of complicated disease, when compared between patients with and without liver test abnormalities. **a** Patients with CRP <16 mg/L; *p* < 0.0001 between patients with and without LTA (log rank). **b** Patients with CRP 16–58 mg/L; *p* = 0.019 between patients with and without LTA (log rank). **c** Patients with CRP >58 mg/L; *p* = 0.101 between patients with and without LTA (log rank)

As CRP was higher in LTA patients, we evaluated the interplay between the elevated CRP and the presence of LTA (Fig. 3a–c). In patients with CRP <16 and 16–58 mg/L, the presence of LTA was associated with a higher prevalence of complicated disease behaviour compared to those without LTA (CRP < 16 mg/L, log rank: *p* < 0.0001; CRP 16–58 mg/L, log rank: *p* = 0.019; CRP > 58 mg/L, log rank: *p* = 0.101).

### Univariate and multivariate analyses

To assess whether LTA is an independent risk factor for developing complicated disease behaviour, we performed univariate (Table 2) and multivariate analyses. The univariate analysis identified the presence of LTA, location of disease, and CRP as risk factors for developing complicated disease behaviour.

**Table 2:** Univariate analysis for the LTA and development of complicated disease behaviour and hospitalizations

	Complicated disease behaviour			Hospitalization		
	HR	95% CI	*p* value	HR	95% CI	*p* value
LTA	2.45	1.6–3.8	<0.0001	1.6	1.1–2.1	0.007
Age at diagnosis			0.92			
A1				Ref		
A2				0.55	0.34–0.89	0.015
A3				0.57	0.34–0.95	0.030
BMI			0.95			
<18				Ref		
18–26				0.67	0.42–1.1	0.102
>26				0.47	0.26–0.86	0.014
Location						0.43
Colonic	Ref					
Ileal only	1.9	1.005–3.7	0.048			
Ileocolonic	1.7	0.91–3.2	0.096			
Upper GI	5.1	1.2–5.1	0.031			
Behavior			0.19			
B1				Ref		
B2				1.6	1.2–2.4	0.006
B3				1.7	0.97–2.9	0.07
Perianal disease			0.39			0.9
Smoking			0.53			0.9
CRP at diagnosis	1.003	1.001–1.006	0.010			0.238
Sex			0.71	1.3	0.9–1.8	0.095

A1 = age at diagnosis <17 years; A2 = age at diagnosis between 17 and 40 years; A3 = age at diagnosis >40 years; B1 = non-stricturing, non-penetrating disease; B2 = stricturing disease; B3 = penetrating disease

*Ref* reference, *LTA* liver test abnormalities, *CRP* C-reactive protein

The multivariate analysis showed that the presence of LTA was associated with a higher risk of developing complicated disease behaviour, as were ileal location and isolated upper disease, whereas CRP at diagnosis was not (Table 3).

**Table 3:** Multivariate analysis for the LTA and development of complicated disease behaviour

	HR	95% CI	*p* value
LTA	2.6	1.5–4.2	<0.0001
Location			
Colonic	Ref		
Ileal only	2.4	1.1–5.1	0.022
Ileocolonic	1.9	0.95–3.9	0.69
Upper GI	6.6	1.2–26.2	0.032
CRP at diagnosis	1.004	1.001–1.007	0.19

*LTA* liver test abnormalities, *CRP* C-reactive protein, *Ref* reference

Using the multivariate analysis, the presence of LTA (HR 1.8, *p* = 0.008, 95% CI 1.2–2.7) and the presence of B2 behaviour (HR 1.9, *p* = 0.028, 95% CI 1.1–3.1) were found to increase the risk of hospitalizations. Patient age at diagnosis >17 years reduced the risk of hospitalization (A2: *p* = 0.033, HR 0.32, 95% CI 0.24–0.94; A3: *p* = 0.005, HR 0.32, 95% CI 0.14–0.70). B3 behaviour (*p* = 0.09), sex (*p* = 0.16), and BMI (*p* = 0.52) did not influence the risk of hospitalization.

For surgery, when all patients were analysed, the presence of complicated disease behaviour at diagnosis (B2: HR 3.6, 95% CI 2.3–5.8, *p* < 0.0001; B3: HR 5.5, 95% CI 2.0–10.7, *p* < 0.0001) was identified as a risk factor.

However, when patients with complicated disease behaviour at diagnosis were excluded, the presence of LTA (*p* = 0.015, HR 2.3, 95% CI 1.17–4.32) and either ileal (*p* = 0.019, HR 2.99, 95% CI 1.2–7.45) or upper GI location (*p* = 0.037, HR 9.57, 95% CI 1.148–79.3) were associated with a higher risk of surgery.

### Sensitivity analysis

A number of sensitivity analyses were performed: when only patients with a normal CRP (below 5 mg/L; *n* = 27) were included, the result of the Kaplan-Meier (KM) analysis for the presence of LTA was similar to that of the analysis with all patients included. When only patients with B1 behaviour were included (inflammatory disease only; *n* = 281), the results of the KM LR and Cox regression analyses were similar to those of the entire cohort. When only patients from the HAGA hospital were analysed (*n* = 225), the results for the KM analysis and Cox regression analysis were similar to those of the entire cohort. When CRP was added to the Cox multivariate model divided into three equal groups instead of as a continuous variable, this did not alter the results (LTA: *p* < 0.0001, HR 2.4; locations L1 and L4: *p* = 0.022, HR 2.42, and *p* = 0.032, HR 5.5, respectively).

## Discussion

In this study of 383 newly diagnosed Crohn’s disease patients, patients with liver test abnormalities more often developed complicated disease behaviour and more often were in need of hospitalization or surgery within 5 years of diagnosis than patients without LTA.

LTAs were present in 34.1% of patients. Most LTAs were mild (less than two times the ULN), and in most instances, only one liver test was elevated. The prevalence of 34% corresponds to the 29% found by Mendes et al. [2] with the prevalence in the literature varying between 3 and 50% [10]. As liver test abnormalities are often ascribed to medication (such as thiopurines) [3], we only included patients with IBD prior to the initiation of medication. The prevalence of chronic liver disease was low: only one patient was excluded due to small duct PSC, and the prevalence of steatosis diagnosed by ultrasound was 6%, similar to a recent paper demonstrating a prevalence of 8.2% in IBD patients [11].

In coeliac disease, a relationship was found between the presence of liver test abnormalities and disease activity. For instance, Zanini et al. found a correlation between the degree of mucosal inflammation, graded according to the Marsh-Oberhuber classification, and elevation of AST, ALT, and GGT, whereas AP was not reported in [5]. In our study of patients with active disease, the prevalence of LTA was highest in patients with the highest CRP (48% when CRP >58 mg/L) and this is in line with what is observed in patients with coeliac disease.

In Crohn’s disease, the relationship between inflammation and development of complications is well established. For instance, Allez et al. demonstrated that patients with more severe inflammation had a higher risk of fistulising disease [6]. In our study, 27% of CD patients developed a new episode of complicated disease behaviour within the first 5 years after diagnosis: 33% in patients with LTA and 14.6% in patients without LTA (*p* < 0.0001). Using the KM log-rank survival analysis, we showed that the presence of LTA was associated with an increased prevalence of complicated disease behaviour, as well as with increased need for hospitalizations.

As the mean CRP was higher in patients with LTA, we analysed whether the increased prevalence of complications in LTA patients was only reflective of the higher CRP concentration in this group. However, as shown in Fig. 3a, patients with a CRP <16 mg/L but with LTA had a higher risk of developing complicated disease compared to those without LTA. This demonstrates that the presence of LTA does not merely reflect a higher CRP concentration but may be a more sensitive indicator of an increased risk of complicated disease behaviour than CRP.

Additional evidence for the independent role of LTA as a risk factor for complicated disease behaviour comes from the multivariate analysis. In the Cox regression analysis, the presence of LTA, as well as isolated ileal disease, and the presence of upper GI disease are independent risk factors for complicated disease behaviour. Additionally, the presence of LTA was independently associated with an increased risk of hospitalizations (HR 1.7, *p* = 0.023) as well as surgery (HR 2.3, *p* = 0.015). For both these outcomes, a relationship with degree of inflammation has been suggested [12, 13].

Although the relationship between inflammation and liver test abnormalities has previously been found in ulcerative colitis [1, 14, 15], this has, to our knowledge, not been demonstrated previously in CD patients. In UC patients, Riegler et al. found a higher prevalence of LTA predominantly in those patients with a higher level of symptoms [14] and Morsy and Hasanain found a relation between a higher endoscopic index of severity and an elevated level of AP, ALT, and AST [15]. In these papers, no follow-up data was available and the mechanism behind this relationship was not explored in these papers.

Why the presence of LTA is associated with complicated disease behaviour is uncertain. But, as gut-liver crosstalk has received much attention recently [16], a number of theories have been suggested: increased intestinal permeability in active CD may lead to exposure of the liver to toxins or antigens of intestinal origin [5]. In coeliac disease, a correlation has been observed between transaminase level and the intestinal permeability index [17]. Coeliac disease patients with more severe inflammation have a higher intestinal permeability, and this is reflected in the higher prevalence of LTA. This may also be the case in CD patients as increased permeability is a hallmark of inflammation in CD and is associated with a more severe disease course [5].

Secondly, enterohepatic circulation of lymphocytes may play a role, in which memory cells from the gut mucosa are recruited to the liver in response to aberrantly expressed gut-homing molecules [18]. When these lymphocytes encounter an intestinal antigen in the liver, immune activation occurs leading to inflammation [18]. The combination of increased permeability and the presence of gut-derived lymphocytes may cause liver test abnormalities in patients with active Crohn’s disease.

We rejected hesitation to use immunomodulatory or biologicals in LTA patients as a potential explanation, as these medications may also induce liver test abnormalities, given the fact that the use of these drugs was similar in LTA and non-LTA patients.

The presence of perianal disease at diagnosis was not related to complicated disease behaviour, in contrast to a number of studies [19, 20]. However, 72% of patients with perianal disease at diagnosis were started on anti-TNF compared to 48% patients without perianal disease (*p* = 0.005). Recently, Magro et al. demonstrated that early treatment with either a biological or an immunomodulator can prevent progression to complicated disease behaviour [21]. The high frequency of anti-TNF usage in patients with perianal disease may have prevented progression to complicated luminal disease. Upper or isolated ileal location of disease was associated with a higher risk of surgery, as demonstrated previously [22, 23]. When all patients were analysed, the patient with complicated disease at diagnosis were also at an increased risk of surgery (stricturing disease: HR 3.6; penetrating: HR 5.5), confirming earlier reports [22, 23]. The presence of LTA was associated with an increased risk of surgery only in those patients without complicated disease behaviour at diagnosis.

In addition to their clinical relevance, our findings appear to underline the importance of the gut-liver axis and emphasize the need for a better understanding of its molecular and genetic components. The limitations of our study are its retrospective nature and the risk of referral bias. We sought to minimize the latter by including only newly diagnosed CD patients and patients from both a tertiary and two large teaching hospitals. The retrospective nature means that the work-up of patients with LTA was different in each case. However, in patients with persistent LTA or in those with higher elevations of liver tests, a more thorough evaluation was always performed, minimizing the chance of missed chronic liver disease in those patients.

In conclusion, the presence of LTA was an independent risk factor for developing complicated disease, need for hospitalization, and need for surgery in this retrospective cohort study. Liver tests may be a widely available, low-cost instrument that helps in identifying high-risk patients who could benefit from a top-down therapeutic regimen.

## Electronic supplementary material

Suppl. Table 4 (DOCX 14 kb)

Suppl. Table 5 (DOCX 13 kb)

Suppl. Table 6 (DOCX 14 kb)
