# Supplementary material for: Liver test abnormalities predict complicated disease behaviour in patients with newly diagnosed Crohn’s disease
Source: Int J Colorectal Dis. 2016 Nov 29;32(4):459–67. doi: 10.1007/s00384-016-2706-3 (PMC5355514; doi:10.1007/s00384-016-2706-3)
Supplement: Supplementary file 1 — (DOCX 14 kb) [file 384_2016_2706_MOESM1_ESM.docx]

**Supplementary tables**

| **Suppl. table 4. Test results in patients with and without LTA** | | | |
| --- | --- | --- | --- |
|  | **All Patients (n=383)** | **LTA (n=131)** | **No LTA (n=252)** |
| **AP** (AP/ULN; IQR) | 0.80 (0.59-0.9) | 1.1 (0.75-1.25)* | 0.67 (0.55-0.79)* |
| **GGT** (GGT/ULN; IQR) | 0.84 (0.36-0.83) | 1.51 (0.69-1.92)* | 0.48(0.31-0.63)* |
| **AST** (AST/ULN; IQR) | 0.60 (0.33-0.65) | 1.03 (0.53-1.19)* | 0.51 (0.33-0.47)* |
| **ALT** (ALT/ULN; IQR) | 0.67 (0.29-0.71) | 1.22 (0.44-1.24)* | 0.43 (0.28-0.50)* |
| **Liver US performed** | 248 (64.8%) | 85 (64.9%) | 163 (64.7%) |
| **Liver steatosis** | 16/248 (6.3%) | 8 (9.3%) | 8 (4.8%) |
| **Biliary stones or sludge** | 19/248 (6.7%) | 5 (4.7%) | 14(7.8%) |
| **Other findings:**  Hemangioma or cysts  Hepatomegaly  Gallbladder polyp or adenomyomatosis | 10/248  3/248  3/248 | 4 (7.1%)  2 (2.4%)  1 (1.2%) | 6 (2.5%)  1 (0.6%)  2 (1.2%) |
| **Viral evaluation** | 185 (48.2%) | 70 (53.4%) | 115 (45.6%) |
| **Auto-immune evaluation** | 85 (22.2%) | 35 (34.2%) | 50 (19.8%) |
| LTA: liver test abnormalities, AP: Alkaline Phosphatase, GGT: Gamma-Glutamyl Transpeptidase, AST: Aspartate aminotransferase, ALT: Alanine Aminotransferase, ULN: upper limit of normal  *p<0.0001 for LTA vs no LTA | | | |
