# Supplementary material for: Liver test abnormalities predict complicated disease behaviour in patients with newly diagnosed Crohn’s disease
Source: Int J Colorectal Dis. 2016 Nov 29;32(4):459–67. doi: 10.1007/s00384-016-2706-3 (PMC5355514; doi:10.1007/s00384-016-2706-3)
Supplement: Supplementary file 2 — (DOCX 13 kb) [file 384_2016_2706_MOESM2_ESM.docx]

| **Suppl. table 5. Relationship between number of elevated liver tests and proportion of patients that develop complicated disease behaviour or is hospitalized.** | | |
| --- | --- | --- |
| **No. of liver tests elevated** | **Complicated disease behaviour*** | **Hospitalization**** |
| **0 (n=252)** | 41 (16.3%) | 95 (37.70%) |
| **1 (n=71)** | 22 (31.0%) | 37 (52.1%) |
| **2 (n=27)** | 10 (37.0%) | 15 (55.63%) |
| **3 (n=22)** | 5 (22.7%) | 7 (31.80%) |
| **4 (n=11)** | 3 (27.3%) | 7 (63.3.6%) |
| *denotes p=0.017 across groups, **denotes p=0.041 across groups. | | |
