# Supplementary material for: Liver test abnormalities predict complicated disease behaviour in patients with newly diagnosed Crohn’s disease
Source: Int J Colorectal Dis. 2016 Nov 29;32(4):459–67. doi: 10.1007/s00384-016-2706-3 (PMC5355514; doi:10.1007/s00384-016-2706-3)
Supplement: Supplementary file 3 — (DOCX 14 kb) [file 384_2016_2706_MOESM3_ESM.docx]

| **Suppl. table 6. Effects of different liver enzymes on complicated behaviour, hospitalizations and surgery** | | | | | | |
| --- | --- | --- | --- | --- | --- | --- |
| **Liver test elevated** | **Complicated disease behaviour** | | **Hospitalization** | | **Surgery** | |
|  | Elevated test | not elevated | Elevated test | not elevated | Elevated test | Not elevated |
| **AP** n= 74 (19.3%) | **25 (33.8%)*** | 56 (8.1%)* | **39 (52.7%)*** | 122 (39.5%)* | 21/81 (28.4%) | 68 (22.0%) |
| **GGT** n=80 (20.9%) | 21 (26.3%) | 6 (19.8%) | 38 (47.5%) | 123 (40.6%) | 21 (26.2%) | 68 (22.4%) |
| **AST** n=35 (9.1%) | 11 (31.4%) | 70 (20.1%) | 18 (51.4%) | 143 (41.1) | 9 (25.7%) | 80 (23.0%) |
| **ALT** n=46 (9.7%) | 12 (26.1%) | 69 (20.5%) | 21 (45.7%) | 140 (41.5%) | 9 (19.6%) | 80 (23.7%) |
| AP: Alkaline Phosphatase , GGT: Gamma-Glutamyl Transpeptidase, AST: Aspartate aminotransferase, ALT: Alanine Aminotransferase  *denotes p<0.05 for patients with vs patients without given abnormal test | | | | | | |
